# Partitioning between recoding and termination at a stop codon–selenocysteine insertion sequence

**DOI:** 10.1093/nar/gkv558

**Published:** 2015-06-03

**Authors:** Suresh Babu Kotini, Frank Peske, Marina V. Rodnina

**Affiliations:** Department of Physical Biochemistry, Max Planck Institute for Biophysical Chemistry, Am Fassberg 11, 37077 Goettingen, Germany

## Abstract

Selenocysteine (Sec) is inserted into proteins by recoding a UGA stop codon followed by a selenocysteine insertion sequence (SECIS). UGA recoding by the Sec machinery is believed to be very inefficient owing to RF2-mediated termination at UGA. Here we show that recoding efficiency *in vivo* is 30–40% independently of the cell growth rate. Efficient recoding requires sufficient selenium concentrations in the medium. RF2 is an unexpectedly poor competitor of Sec. We recapitulate the major characteristics of SECIS-dependent UGA recoding *in vitro* using a fragment of *fdhF*-mRNA encoding a natural bacterial selenoprotein. Only 40% of actively translating ribosomes that reach the UGA codon insert Sec, even in the absence of RF2, suggesting that the capacity to insert Sec into proteins is inherently limited. RF2 does not compete with the Sec incorporation machinery; rather, it terminates translation on those ribosomes that failed to incorporate Sec. The data suggest a model in which early recruitment of Sec-tRNA^Sec^–SelB–GTP to the SECIS blocks the access of RF2 to the stop codon, thereby prioritizing recoding over termination at Sec-dedicated stop codons.

## INTRODUCTION

Selenocysteine (Sec) is the 21st genetically encoded amino acid that is incorporated into proteins during protein synthesis on the ribosome (1,2). Selenoproteins are found in bacteria, archaea and many eukaryotes (3–7). Sec is structurally similar to cysteine (Cys), with a selenium atom at the place of the sulfur, forming a selenol group. The higher chemical reactivity of Sec compared to Cys (8) explains why Sec is mostly found at the active center of enzymes that catalyze oxidation-reduction reactions (9,10). Unlike the 20 standard amino acids which are encoded by their specific sense codons, Sec is encoded by a stop codon, UGA, which normally serves as a signal for the termination of protein synthesis. In bacteria, the specific incorporation of Sec into proteins requires a selenocysteine insertion sequence (SECIS) in the mRNA, a stem-loop structure located immediately downstream of the in-frame UGA codon at which Sec is incorporated (11). Sec delivery to the ribosome requires Sec-specific tRNA^Sec^ and proteins SelA, SelD and SelB. tRNA^Sec^ is first aminoacylated with serine by seryl-tRNA synthetase (SerRS) (12) and then converted to Sec-tRNA^Sec^ by Sec synthase (SelA) using selenophosphate as the selenium donor (13). Selenophosphate is produced from selenide by selenophosphate synthase SelD at the cost of ATP (14). Sec-tRNA^Sec^ is delivered to the ribosome by the specialized elongation factor SelB, a GTP-binding protein that belongs to the family of translational GTPases (other family members are elongation factors Tu and G, initiation factor 2, release factor 3 and their eukaryotic homologs) and is evolutionary closely related to translation initiation factor 2 (eIF2γ) (15). Sequence comparisons and a homology model of SelB based on the structure of EF-Tu, the general aminoacyl-tRNA delivery factor, suggested that SelB consists of four domains (16). The N-terminal domains 1, 2 and 3 are similar to the corresponding domains of EF-Tu (17). They provide the binding site for GTP/GDP (domain 1) and Sec-tRNA^Sec^. The C-terminal domain 4 of SelB does not show any sequence or structural similarity to the known translational factors; domain 4 recognizes the SECIS (18).

The affinity of SelB–GTP for Sec-tRNA^Sec^ is very high, with a *K*_d_ in the picomolar range (19). SelB binding to the SECIS is rapid (*k*_on_ = 10^8^ M^−1^ s^−1^) and tight (*K*_d_ in the nanomolar range (20)). These high affinities suggest that in the cell the Sec-tRNA^Sec^–SelB–GTP complex binds to the SECIS before it enters the ribosome, thereby facilitating the recruitment of Sec-tRNA^Sec^ to the UGA codon preceding the SECIS. Nevertheless, the efficiency of UGA recoding into Sec by Sec-tRNA^Sec^–SelB–GTP in *Escherichia coli* is believed to be very low, ∼5% (21), at least when the cells are rapidly growing in rich media (22). Sec incorporation remained low, ∼10%, when the individual components of the Sec-insertion machinery (SelB, tRNA^Sec^ and SelA) were overexpressed (21). However, in other reports, the estimated Sec insertion efficiency was higher, ∼25% in rich medium, and reached ∼60% at conditions of slow growth (22). Overexpression of release factor 2 (RF2), which normally reads the UGA codon and promotes translation termination, only moderately (<2 times) decreased the Sec incorporation efficiency, which was interpreted in terms of direct competition of the Sec-tRNA^Sec^–SelB–GTP complex and RF2 for binding to the UGA codon (22).

The reasons for the low Sec insertion efficiency and the presumed growth-dependent variations are not clear. Genetic analysis indicated that the low efficiency is caused by termination at the UGA codon rather than by the presence of a (stable) SECIS hairpin structure or the competition of the bulk of EF-Tu ternary complexes with SelB–GTP–Sec-tRNA^Sec^ (21,22). Potentially, RF2 can compete with Sec-tRNA^Sec^ for binding to a UGA codon, resulting in premature termination on a fraction of ribosomes. As the cell growth rate decreases, the production of the overall number of RF2 molecules in the cell is reduced (although apparently not their free concentration) (23) and, at slow cell growth, the selection rate of Sec-tRNA^Sec^ may exceed the RF2 selection rate (22), whereas the concentration of SelB remains constant (24). It remains unclear how this presumed ‘dynamic competition’ should work if the concentration of RF2 does not change with the growth rate. The discrepancies in the estimations for the Sec insertion efficiency and the lack of a conclusive model for the competition between RF2 and Sec-tRNA^Sec^ at the UGA codon prompted us to re-visit the competition model *in vivo* using a dual-luciferase reporter assay and *in vitro* using a fully reconstituted translation system synthesizing a fragment of the bacterial selenoprotein formate dehydrogenase H (FdhH, product of the *fdhF* gene). The results provide an insight into the cellular strategies to achieve efficient reassignment of the stop codon and minimize premature termination by release factors.

## MATERIALS AND METHODS

### Buffers and reagents

The experiments were carried out in buffer A (50 mM Tris-HCl, pH 7.5, 70 mM NH_4_Cl, 30 mM KCl, 3.5 mM MgCl_2_, 8 mM putrescine, 10 mM DTT) at 37°C. Chemicals were from Roche Molecular Biochemicals, Sigma Aldrich or Merck. Radioactive compounds were from Hartmann Analytics.

### Vectors

**Figure 1.**
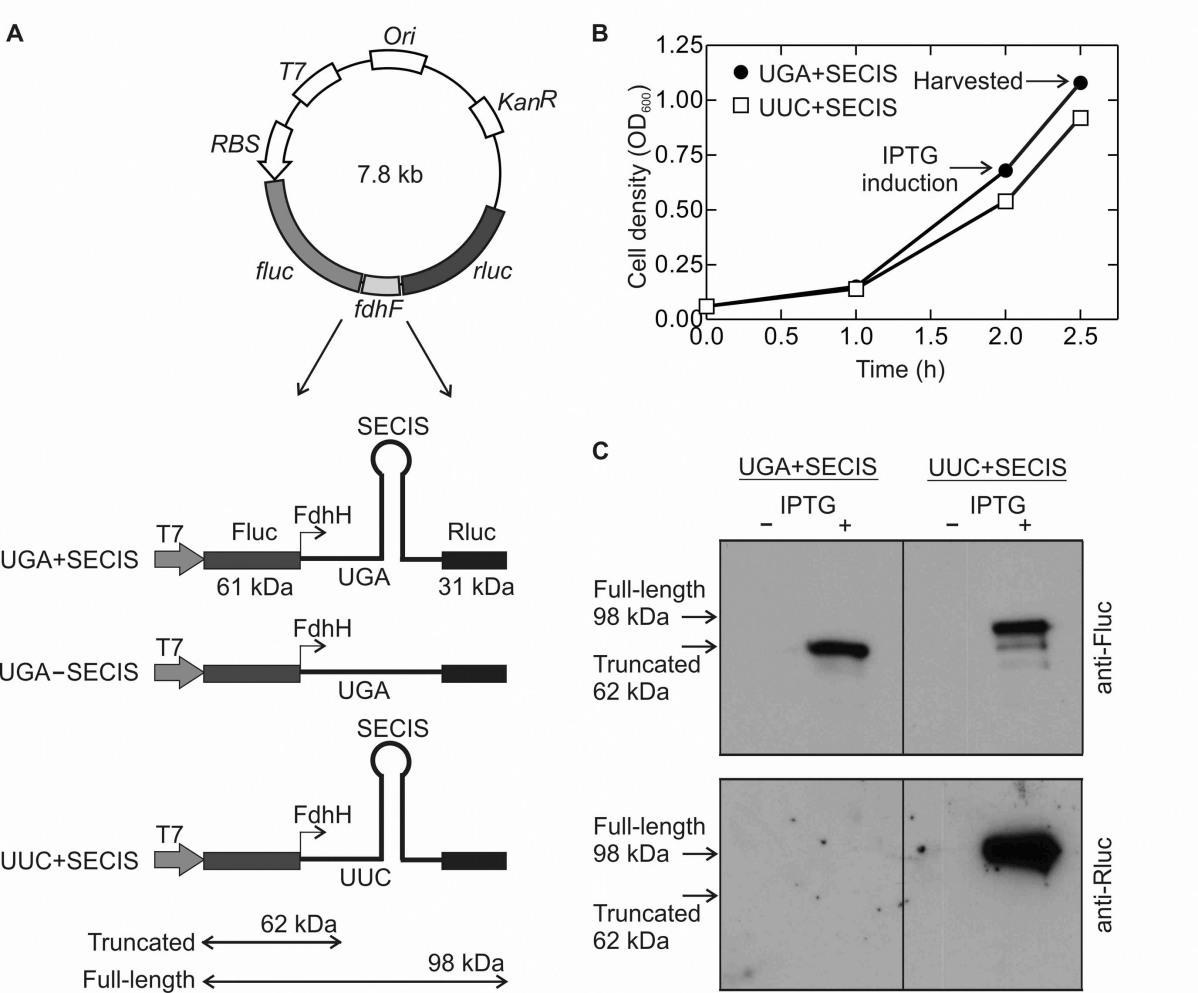
Experimental system to investigate UGA recoding by Sec. (**A**) Constructs for dual-luciferase reporter assay. A fragment of the *fdhF* gene was inserted between firefly (*fluc*) and renilla (*rluc*) luciferase genes. The fragment of the wild-type *fdhF* gene codes for amino acids 130–179 including a UGA codon at position 140 followed by the SECIS. Test constructs: recoding segment containing both the stop codon and the SECIS (UGA+SECIS), or without the SECIS element (UGA–SECIS), or with the UGA codon replaced with a UUC codon (coding for Phe) (UUC+SECIS). (**B**) Growth curves of *E. coli* Tuner (DE3) cells transformed with test constructs. Protein expression was induced by IPTG addition. Times of protein expression induction (initial-log phase) and harvest are indicated. (**C**) Expression of the Fluc–FdhF (62 kDa) and Fluc–FdhF–Rluc (98 kDa) proteins was visualized by western blot analysis using antibodies against Fluc (upper panel) or Rluc (lower panel). Fluc–FdhF–Rluc is synthesized as a result of UGA or UUC read-through.

Vectors used for *in vivo* experiments contained Fluc and Rluc genes amplified by polymerase chain reaction (PCR) from vectors pGEM-luc and pRL (Promega), respectively, and ligated into pET24a(+) (Novagen) (25). A fragment of the *E. coli fdhF* gene coding for amino acids 130–179 (Sec_140_) was inserted between the two luciferase genes (Figure 1A). All other constructs were generated by introducing point mutations or deletions using PCR. For RF2 competition experiments, the *E. coli prfb* gene coding for RF2 was cloned into pETcoco-1 (Novagen), a C-terminal His-tag was added and 0-reading frame ensured by deletion of a T at the native +1 frameshifting site to increase expression (Figure 3A). The RF2 APA construct was generated by PCR.

**Figure 2.**
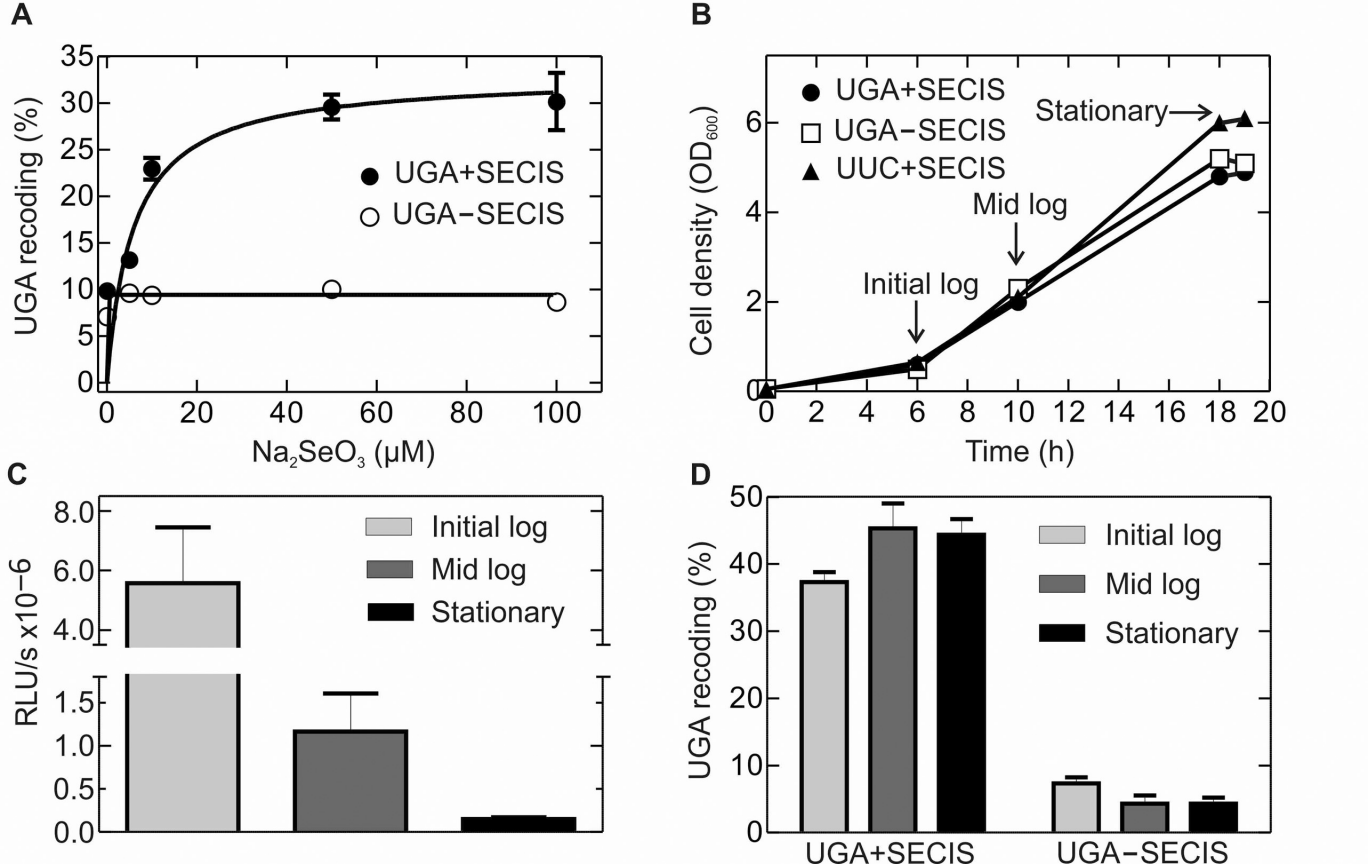
Dependence of UGA read-through on selenium source concentration and cell growth conditions. (**A**) Increase in UGA read-through with selenium supply. Cells were grown in TPG medium supplemented with different concentrations of Na_2_SeO_3_ as indicated. The efficiency of Sec incorporation was measured by comparing the luciferase activity ratios of Rluc/Fluc in constructs containing the UGA and UUC codons preceding the SECIS (Materials and Methods). (**B**) Growth curves of *E. coli* Tuner (DE3) cells transformed with test constructs in TPG medium containing 50 μM Na_2_SeO_3_. Protein expression was induced by IPTG addition. Times of protein expression induction are indicated (arrows). (**C**) Fluc activity at different growth rates as an indicator for protein expression given in relative luciferase units per min (RLU/s). (**D**) UGA-recoding efficiency at different growth rates calculated as in A.

**Figure 3.**
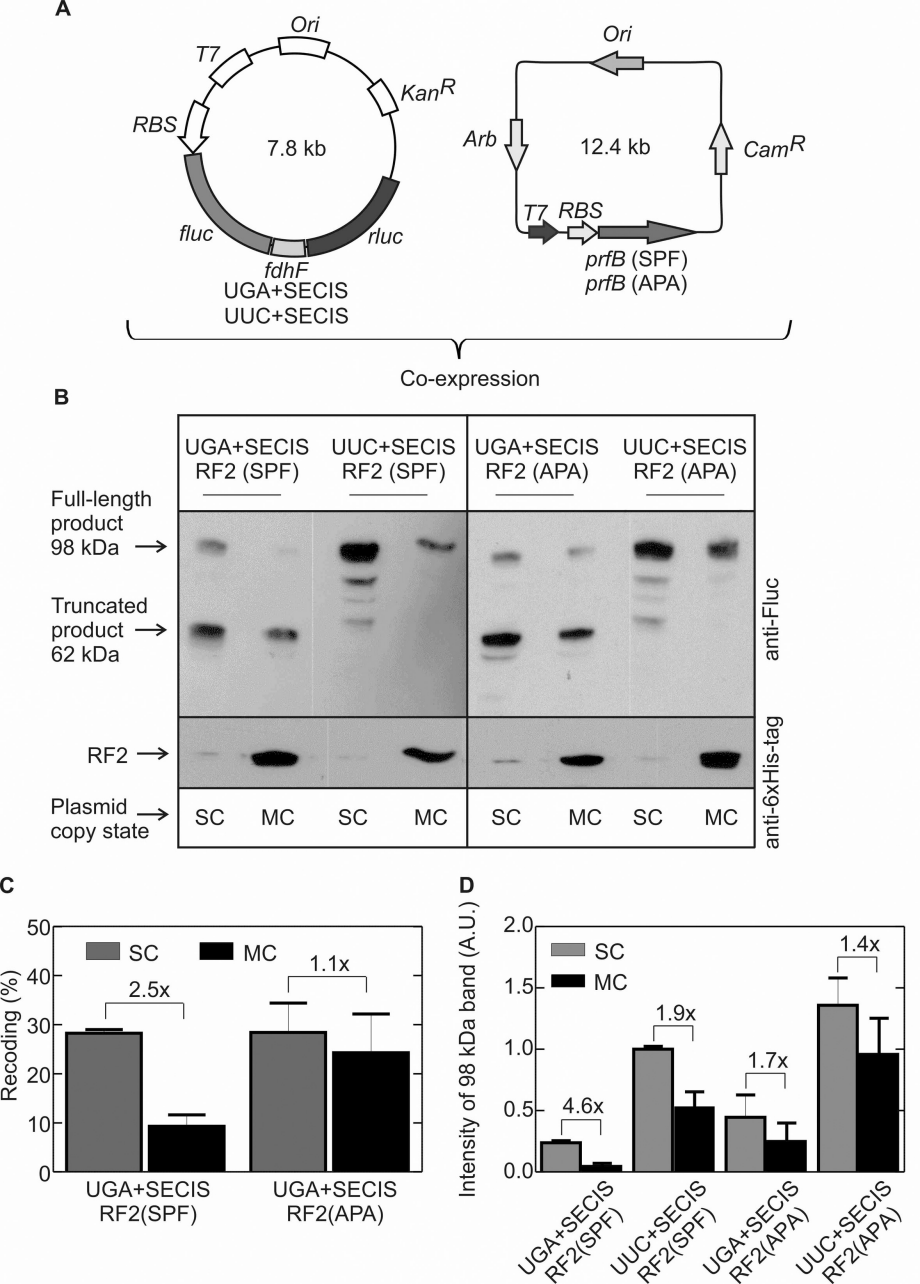
Effect of RF2 on Sec insertion *in vivo*. (**A**) Co-expression of Fluc–FdhF–Rluc and RF2 constructs. *prf*(SPF) is a construct for 0-frame expression of RF2 with the native stop-codon recognition motif (SPF). In the *prf*(APA) construct, the SPF motif is mutated to APA to produce RF2 that is inactive in translation termination. (**B**) Example of a sodium dodecyl sulphate-polyacrylamide gel electrophoresis separation of the Fluc–FdhF–Rluc expression products in the presence of RF2 (SPF or APA) obtained from a single-copy (SC) or multi-copy (MC) plasmids. Fluc-FdhF and Fluc–FdhF–Rluc products were visualized by western blot analysis using an anti-Fluc antibody. The identity of the smaller peptides observed with the UUC+SECIS construct is not known. The amounts of RF2 in the same samples were detected using an antibody against the His-tag of RF2 (see also Supplementary Figure S1). (**C**) UGA recoding analysis from the western blot band intensity. Recoding was calculated as the ratio of the read-through band over the sum of the read-through and truncated bands. Error bars represent SEM from two experiments. The extent of read-through obtained with the UUC+SECIS construct defines 100% read-through efficiency. (**D**) Direct comparison of the read-through band intensities for all constructs. The band intensity for the product of UUC+SECIS translation at the SC RF2 conditions was taken as 1. Error bars represent SEM from two independent experiments.

### Expression of Fluc–FdhF–Rluc fusion proteins

Initial experiments (Figure 1) were carried out in LB medium containing kanamycin (30 μg/ml) and no added selenium source. All other expression experiments were done in TPG medium (1% tryptone, 100 mM potassium phosphate, pH 6.5, 0.5% glycerol, 1 mM MgSO_4_, 0.1 mM CaCl_2_, 0.4 μM H_3_BO_3_, 30 nM CoCl_2_, 10 nM CuSO_4_, 10 nM ZnSO_4_, 80 nM MnCl_2_, 10 μM FeCl_3_ and 50 μM Na_2_SeO_3_), if not stated otherwise. Dual-luciferase constructs were transformed into *E. coli* Tuner (DE3) cells (Novagen), cultures were inoculated from single colonies, and grown at 37°C until OD_600_ reached 0.5–0.7 (Figures 1 and 2A) or for growth-phase experiments until OD_600_ reached 0.5–0.7 (initial-log phase), 2–2.5 (mid-log phase) and 5–6 (stationary phase) (Figure 2B). Expression was induced by the addition of IPTG (1 mM) and growth continued for 30 min. Cells were harvested and stored at −80°C.

For RF2 co-expression experiments (Figure 3), dual reporter plasmids and RF2 coding pETcoco-1 plasmids were co-transformed into *E. coli* Tuner (DE3), cultures were inoculated from single colonies, and grown in TPG medium supplemented with kanamycin (30 μg/ml) and chloramphenicol (25 μg/ml) at 37°C. To maintain the single-copy (SC) state of the pETcoco-1 constructs, the medium contained glucose (0.2%); to induce the multi-copy (MC) state, L-arabinose (0.01%) was added. Cells were grown up to OD_600_ = 0.5–0.7, expression was induced by IPTG (1 mM) and cells were further grown for 30 min, harvested and stored at -80°C.

### Western blotting

0.5 OD_600_ of cell extracts were boiled in SDS sample buffer and samples were separated on 12% SDS polyacrylamide gel. Membranes were blocked in 1 x PBS containing 20% skim milk powder and 0.1% Tween 20 overnight at 4°C. To detect Fluc, membranes were incubated with a goat primary anti-luciferase antibody pAb IgG (Promega) (1:1000) for 2 h at room temperature, washed three times with 1 x PBS containing 0.1% Tween 20 (1 x PBST), and then for 45 min at room temperature with secondary antibody Anti-Goat HRP (1:10 000). Same membranes were stripped by washing three times with 1 x PBST and used to detect Rluc using mouse primary antibody anti-Rluc IgG (1:10 000) (Millipore). After incubation for 2 h at room temperature and washing three times with 1 x PBST, membranes were incubated for 1 h with secondary antibody anti-mouse IgG (1:5000) (Jackson ImmunoResearch) raised in goat. For detection of RF2-His-tag, antibodies against-His-tag were used to visualize the protein on the same membrane as Fluc and Rluc (Figure 3B). The antigen–antibody complexes were detected using Super Signal West Pico Chemiluminescence Substrate (Pierce). Membranes were exposed to high performance chemiluminescence film (GE Healthcare) for 10 s and films were developed using standard procedures. Western blot band intensities were quantified using MultiGauge software (Fujifilm).

### Luciferase activity measurements

Cells were resuspended in lysis buffer (10 mM Tris-HCl, pH 8.0, 1 mM EDTA, 5 mg/ml lysozyme, 70 μl of lysis buffer per OD_600_ unit) and lysed on ice for 10 min and then incubated for 15 min at 37°C. Cell debris was removed by centrifugation for 5 min at 10 000 g. Fluc and Rluc activities were measured separately in a luminometer (Sirius Single, Berthold) with a delay time of 2 s and an integration time of 5 s. To measure Fluc activity, 5 μl of supernatant was mixed with 100 μl of Beetle Juice (PJK GmbH) at room temperature. Rluc activity was measured by mixing 5 μl of cell extract with 100 μl of Renilla Glow Juice (PJK GmbH) at room temperature. Setting the ratio of Rluc and Fluc for the construct containing a UUC codon and the SECIS (control) as 100%, recoding efficiencies were calculated as following: ((Rluc(test)/Fluc(test))/((Rluc(control)/Fluc(control)) x 100%.

### *In vitro* transcription and purification of model *fdhF* mRNAs

The short model mRNA (mLP75s) construct was described previously (26) (Supplementary Figure S2A). The model mRNA comprising a fragment of *fdhF* gene (amino amino acids 130–156) was custom synthesized and inserted into the *in vitro* transcription vector pBluescript II SK (+) (Eurofins). *In vitro* transcription reactions were performed and mRNAs purified as described (26).

### Ribosomes, tRNAs and translation factors

Ribosomes, translation factors and tRNAs were from *E. coli*. Ribosomes from *E. coli* MRE 600, fMet-tRNA^fMet^, f[^3^H]Met-tRNA^fMet^, [^14^C]Phe-tRNA^Phe^, EF-Tu, EF-G, initiation factors and [^3^H]Sec-tRNA^Sec^ were prepared as described (19,27–31). Total tRNA from *E. coli* MRE 600 was purchased from Roche Diagnostics. Plasmids coding for *E. coli* SelA and SelD were from M. Wahl (Free University of Berlin). The plasmid coding for *E. coli* SelB, SelC, (32) was a kind gift from A. Böck (Ludwig Maximilian University, Munich). SelA was purified as described (33). 6-His-tagged SelD and SerRS were purified on Ni-NTA agarose (Qiagen) according to the manufacturer's protocol. RF2 was prepared as described (34). Sec-tRNA^Sec^ was prepared and purified as described (19). Aminoacylation of total tRNA from *E. coli* MRE600 (Roche) was carried out using amino acids Thr, Asn Cys, Asp, Ala, Arg and Val (0.3 mM each), S100 (6%) and ATP (3 mM) in buffer A at 37°C for 30 min. Where necessary, non-labeled Val was replaced with [^14^C]Val (25 μM). Aa-tRNA was purified by phenol extraction and ethanol precipitation and purified by fast protein liquid chromatography (FPLC) on a MonoQ column (5 × 50 mm, GE healthcare) according to the standard protocol (35).

### Expression and purification of SelB

The plasmid harboring SelB was transformed into BL21 (DE3) cells. SelB expression and purification were optimized compared to the earlier published protocol (20) to obtain higher purity and full activity of SelB in the formation of the Sec-tRNA^Sec^–SelB–GTP complex. SelB was expressed in LB medium containing ampicillin (100 μg/ml) at 25°C when OD_600_ reaches 0.6–0.7 with the 0.1 mM IPTG. Induction was continued for 6 h at 25°C. Cell pellets were resuspended in buffer C (50 mM Tris-HCl, pH 8.0, 500 mM KCl, 10 mM MgCl_2_, 5 mM 2-mercaptoethanol, 0.1 mg/ml PMSF, 7% glycerol). Cells were opened by lysozyme treatment. After centrifugation, supernatants were diluted 10-fold to bring the KCl concentration to 50 mM and loaded on to Q-Sepharose equilibrated with buffer C with 60 mM KCl. SelB was eluted with a KCl gradient (60–250 mM) and eluted at 150–200 mM KCl. After buffer exchange to buffer D (50 mM K-HEPES pH 6.8, 100 mM KCl, 10 mM MgCl_2_, 5 mM 2-mercaptoethanol, 7% glycerol), SelB was loaded onto SP-Sepharose equilibrated with buffer D. SelB was eluted with a gradient of KCl (100–500 mM) and eluted at 350–450 mM KCl. SelB was re-buffered and stored in buffer E (50 mM K-HEPES pH 7.5, 400 mM KCl, 5 mM MgCl_2_, 5 mM 2-mercaptoethanol, 22% glycerol). Aliquots were frozen in liquid nitrogen and stored at −80°C. The SelB concentration was determined by absorbance at 280 nm (ϵ_280_ = 81 080 M^−1^ cm^−1^).

### *In vitro* translation

Initiation complexes were prepared by incubating 70S ribosomes (1 μM) with a 10-fold excess of *fdhF* mRNA, initiation factors 1, 2 and 3 (1.5 μM each), f[^3^H]Met-tRNA^fMet^ (1.5 μM) and GTP (1 mM) in buffer B (50 mM Tris-HCl, pH 7.5, 70 mM NH_4_Cl, 30 mM KCl, 7 mM MgCl_2_, 1 mM DTT) for 1 h at 37°C. Initiation complexes were purified by centrifugation through 400 μl sucrose cushions (1.1 M sucrose in buffer B) at 259 000 x g for 2 h (RC M120 GX ultracentrifuge, Sorvall). Pellets were dissolved in buffer B to a final concentration of 5 μM, stock frozen in liquid nitrogen and stored at −80°C. To prepare the 70S–fMet-Phe-tRNA^Phe^ complex, initiation was carried out using non-labeled fMet-tRNA^fMet^ as above (without purification), then [^14^C]Phe-tRNA (8 μM), EF-Tu (16 μM), EF-G (0.033 μM), GTP (1 mM), phosphoenol pyruvate (3 mM), pyruvate kinase (0.1 mg/ml) were added and the mixture incubated for 1 min at 37°C. The ribosome complex was purified by centrifugation as described above.

To prepare aa-tRNA–EF-Tu–GTP complexes, EF-Tu (120 μM) was incubated with pyruvate kinase (0.1 mg/ml), phosphoenol pyruvate (3 mM) and GTP (1 mM) in buffer A for 15 min at 37°C. Total aminoacyl-tRNA (60 μM) was added and incubated for 1 min at 37°C. The concentrations of EF-Tu and aminoacyl-tRNAs were optimized to reach the maximum translation yield up to the UGA stop codon. To prepare [^3^H]Sec–SelB–GTP, SelB (5 μM) was incubated with DTT (10 mM) and GTP (3 mM) in buffer A for 5 min at 37°C. An equal amount of [^3^H]Sec-tRNA^Sec^ (5 μM) was added and incubated for 5 min at room temperature. *In vitro* translation was initiated by the addition of EF-G (2 μM), EF-Tu and SelB ternary complexes in the presence or absence of RF2 to the 70S–*mLP75s*–f[^3^H]Met-tRNA^fMet^ or 70S–*fdhF*–fMet-[^14^C]Phe-tRNA^Phe^ complexes. Translation was carried out at 37°C. If not stated otherwise, final concentrations were: ribosomes (0.22 μM), [^3^H]Sec–SelB–GTP (1 μM), aa-tRNA–EF-Tu–GTP complexes (60 μM), EF-G (4 μM), and RF2 as indicated in the figure legends. Translation was stopped by adding EDTA (250 mM), KCl (600 mM) and iodoacetamide (25 mM), and samples were incubated for 20 min at room temperature to alkylate Sec. Then, tRNA was digested by incubation with Tris-base (100 mM, pH > 10) for 1 h at 37°C; samples were neutralized by adding acetic acid. Translation products were analyzed by HPLC on RP8 using an adapted gradient of 0–65% acetonitrile in 0.1% TFA. To analyze peptide release, two different methods were used. When peptide release was studied with the short model mRNA coding for fMet-Stop, the reactions were stopped by the addition of cold 10% TCA, and the amount of intact f[^3^H]Met-tRNA^fMet^ was determined by nitrocellulose filtration of the TCA precipitates (34). When a long peptide product was analyzed after translation of *fdhF* mRNA, translation was stopped and ribosome-bound peptidyl-tRNA set free by treatment with sodium acetate (50 mM) pH 4.5, KCl (500 mM), EDTA (2 mM), DTT (2 mM). The mixture was centrifuged at 259 000 x g for 20 min at 4°C and the supernatant was diluted 10-fold with sodium acetate (50 mM) pH 4.5, MgCl_2_ (1 mM), DTT (2 mM) to bring the KCl concentration to ∼50 mM and then applied on a MonoQ column equilibrated by sodium acetate (50 mM) pH 4.5, MgCl_2_ (1 mM), DTT (2 mM) buffer. Free amino acids and peptides remained in the flow-through, peptidyl- and aa-tRNAs were eluted by a buffer consisting of sodium acetate (50 mM) pH 4.5, MgCl_2_ (1 mM), DTT (2 mM), applying a gradient of 0–1.2 M KCl.

## RESULTS

### Efficiency of UGA recoding *in vivo*

We determined the efficiency of UGA recoding by Sec *in vivo* using a plasmid-encoded dual-luciferase Fluc/Rluc reporter assay following the validated approaches (25,36). A fragment of the wild-type *E. coli* selenoprotein gene *fdhF* was inserted between the genes for firefly (*fluc*) and renilla (*rluc*) luciferases (Figure 1A). The *fdhF* fragment encoded amino acids 130–179, including the stop codon at position 140 (UGA_140_) followed by the SECIS (encoding amino acids 141–153). Expression of Fluc was independent of Sec insertion and was induced by the addition of IPTG. Translation of the *fluc–fdhF–rluc* mRNA required recoding of UGA_140_ by Sec-tRNA^Sec^. As a control for misreading of the UGA codon, which would result from a Sec-independent translation of *rluc*, we used a construct that lacked the SECIS and thus should be impaired in SelB binding. The maximum level of Rluc synthesis was determined by the expression from the control plasmid containing a UUC codon (which directs Phe insertion) in the place of UGA_140_.

We first validated the assay by expressing Fluc and Rluc in cells growing exponentially without an additional selenium source (Figure 1B). Cells were grown at aerobic conditions in LB medium at 37°C, induced by 1 mM IPTG at initial-log phase (OD_600_ ∼0.6–0.7) and harvested 30 min after induction (Figure 1B). After opening the cells, expression of the Fluc–FdhF–Rluc fusion protein was measured. Because our attempts to measure the ratio of luciferase activities in these experiments were unsuccessful, owing to low or irreproducible activity of Rluc when its synthesis required UGA read-through, we used western blotting with anti-Fluc and -Rluc antibodies (Materials and Methods). When no selenium was added to the medium there was essentially no synthesis of Rluc from constructs containing the wild-type *fdhF* (UGA_140_)+SECIS construct (Figure 1C), suggesting that read-through of UGA_140_ was negligible. In contrast, when the UGA_140_ codon was replaced with UUC, Rluc was synthesized efficiently. This indicated that in the absence of added selenium, the amount of Sec-tRNA^Sec^ in the cell was insufficient for UGA recoding.

We then examined the effect of supplementing the medium with Na_2_SeO_3_ as a selenium source at initial-log phase. Under these conditions, we could reliably measure both Fluc and Rluc activities and determine the read-through efficiency from the ratio of Rluc/Fluc after reading the UGA codon relative to the UUC control (Materials and Methods) (Figure 2A). At initial-log phase, Sec insertion reached 30–35% with increasing selenium levels, with half-saturation at *K*_1/2_ = 6 μM. In comparison, UGA read-through in the construct lacking the SECIS element was 5–10%. This relatively high level of apparent read-though may represent the background of the dual-luciferase reporter system, because it is essentially independent of Na_2_SeO_3_ concentration and because western blot analysis shows negligible read-through in the absence of selenium (Figure 1C). To compare the UGA recoding efficiency at various phases of cell growth, i.e. initial-log, mid-log and stationary phase, cells were grown in TPG medium containing 50 μM Na_2_SeO_3_ (Figure 2B). The Fluc activity decreased dramatically with increasing cell density, reflecting the global shutdown of protein synthesis at the stationary phase (Figure 2C). However, the extent of Sec insertion corrected for the decreased total amount of protein was independent of the growth phase: the efficiency of SECIS-directed UGA recoding was ∼40% at any growth phase, compared to ∼5–8% of SECIS-independent read-through (Figure 2D), where 100% corresponds to the decoding efficiency of a sense codon UUC by Phe-tRNA^Phe^. In principle, the incomplete UGA read-through may either reflect the inherent activity of the Sec-tRNA^Sec^–SelB–GTP machinery in UGA decoding, or the competition with RF2 for the stop codon, or interference by EF-Tu complexes with aminoacyl-tRNA. The latter possibility was excluded (21); furthermore, the observed low frequency of UGA misreading in the absence of selenium also argues against the idea that EF-Tu complexes can affect stop-codon reading (Figure 1C). In contrast, an inhibitory effect of RF2 on Sec incorporation is a widely accepted notion, which we sought out to test in the following experiments.

### Does translation termination compete with Sec insertion *in vivo*?

To understand whether RF2 and Sec-tRNA^Sec^–SelB–GTP compete for UGA, we co-expressed various amounts of RF2 with the Fluc–FdhF–Rluc fusion protein during the initial-log growth phase (Figure 3A and B). Normally, expression of wild-type RF2 requires programmed +1 frameshifting, which is feedback-regulated by the amount of RF2 in the cell and is thus difficult to control. To overcome this difficulty, we used an RF2 construct in which the frameshifting site was removed and RF2 was expressed from the 0-reading frame of the mRNA; the plasmid-encoded RF2 contained a 6xHis-tag for detection by anti-His antibodies. The gene encoding for 0-frame RF2 (*prfB*; SPF denotes the wild-type stop-codon recognition motif) was cloned into the pETcoco-1 vector, which allowed us to increase the plasmid copy number from SC to MC by the addition of L-arabinose (Materials and Methods). Quantification of the RF2 expression using anti-6xHis-tag antibodies showed that the expression at MC conditions resulted in a 12-fold increase of the RF2 concentration compared to SC conditions (Supplementary Figure S1); at these conditions, the expression of the chromosomally encoded RF2 is probably shut down by the excess of the plasmid-encoded factor. To determine the read-through efficiency, we used western blot analysis, because (i) they turned out to yield more reproducible values with less background than the dual-luciferase assay and (ii) the amounts of RF2 and of the read-through product could be evaluated by the same technique. When RF2 was expressed at SC conditions, the efficiency of UGA recoding by Sec was ∼30% (Figure 3C), which is similar to Sec incorporation in the presence of endogenous RF2 only (Figure 2D). At MC conditions, i.e. upon a 12-fold increase of RF2 concentration, the efficiency of UGA recoding decreased 2.5-fold. As a control, we used RF2(APA) constructs with an altered stop-codon recognition motif (Figure 3A). In the presence of the mutant factor, which was inactive in termination, the efficiency of UGA recoding was also 30% at SC conditions and 25% at MC conditions (Figure 3C). While the read-through efficiency here was calculated as a ratio of the read-through product (98 kDa) to the sum of the truncated (62 kDa) and read-through products, we also compared the absolute intensities of the full-length product bands at different conditions to monitor potential unspecific effects of RF2 overexpression. In fact, this comparison indicated that part of the RF2 effect was unspecific: also when UGA was replaced by UUC, Rluc expression was reduced by 1.9-fold, suggesting that RF2 expression interferes not only with Sec but also with the incorporation of standard amino acids (Figure 3D). Similarly, the inactive RF2(APA) showed an unspecific 1.4–1.7-fold inhibitory effect on Fluc–FdhH–Rluc expression suggesting that protein overexpression *per se* can decrease the test construct expression. In contrast, the 2.5-fold specific inhibition of Sec insertion by RF2 (Figure 3C) seems quite little for a 12-fold increase in RF2 concentration. Thus, although the efficiency of UGA recoding by Sec is limited to ∼30–40% *in vivo*, it does not appear to correlate directly with the amount of RF2 present in the cell, and thus the competition between Sec-tRNA^Sec^–SelB–GTP and RF2 cannot fully account for the efficiency of UGA recoding.

### UGA recoding *in vitro*

**Figure 4.**
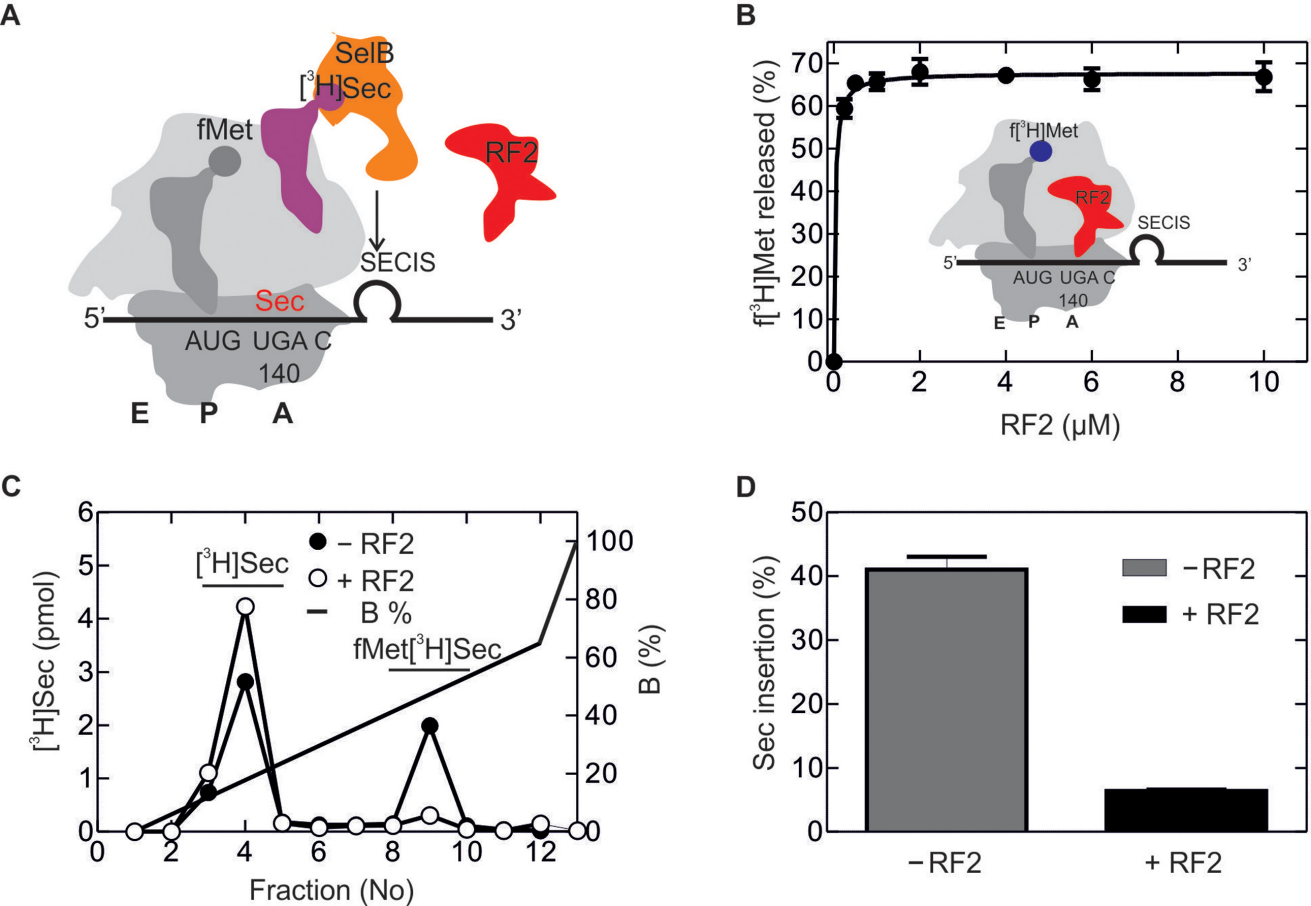
Sec incorporation *in vitro* guided by a short model mRNA. (**A**) Schematic of the experimental setup. Initiation complex formation with the short model mRNA coding for AUG and UGA followed by the SECIS results in the unwinding of the lower part of the SECIS helix. Ribosomal subunits are shown in dark gray (30S) and light gray (50S), respectively; initiator tRNA is shown darker gray, [^3^H]Sec-tRNA^Sec^ is magenta (radio-label indicated), SelB-GTP orange and RF2 red. A, P and E denote the aminoacyl-, peptidyl- and exit sites of the ribosome, respectively. (**B**) f[^3^H]Met release upon addition of increasing amounts of RF2 in the absence of Sec-tRNA^Sec^–SelB–GTP. Inset, schematic of the experiment. To monitor release, radio-labeled f[^3^H]Met-tRNA^fMet^ (radio-label shown blue) was used (34). (**C**) Sec insertion into the dipeptide f[^3^H]MetSec in the absence and presence of RF2 (7 μM). Dipeptide analysis was carried out by HPLC on a RP8 column (Materials and Methods). (**D**) Quantification of the read-through product from the ratio of fMet[^3^H]Sec to total [^3^H]Sec.

To understand the interplay between the termination and recoding machineries, we next sought to establish an *in vitro* translation system that incorporated Sec in proteins with the same efficiency as *in vivo*. We first used a simplified model mRNA (26,37) in which the start codon, AUG, is immediately followed by the UGA stop codon and the SECIS (Supplementary Figure S2A). In the absence of RF2, this model mRNA appears to direct GTP hydrolysis by SelB and Sec incorporation to the same extent as *fdhF* mRNA (37), and thus seemed a suitable model system to start with. f[^3^H]Met-tRNA places the AUG codon in the P site. Because the lower part of the SECIS must unwind to be accommodated in the mRNA tunnel of the 30S subunit, we expect that at this stage only the upper part of the SECIS is still intact (Figure 4A). In the absence of Sec-tRNA^Sec^–SelB–GTP, RF2 promoted efficient termination in this system even at rather low concentrations (Figure 4B). Upon addition of [^3^H]Sec-tRNA^Sec^–SelB–GTP in the absence of RF2, the fMet[^3^H]Sec dipeptide was formed on 40% of ribosomes, indicating that this simplified system is competent in Sec recruitment (Figure 4C and D). However, when RF2 was added, Sec insertion dropped from ∼40 to 6%, thus essentially abolishing UGA decoding by Sec-tRNA^Sec^. While these results show that RF2 could compete with Sec-tRNA^Sec^–SelB–GTP *in vitro*, the extent of inhibition was inconsistent with the high read-through efficiency observed *in vivo* at moderate RF2 concentrations (Figures 2D and 3C). This prompted us to develop a more natural translation system dedicated to the synthesis of the FdhH fragment, similar to that used in the *in vivo* experiments described above.

**Figure 5.**
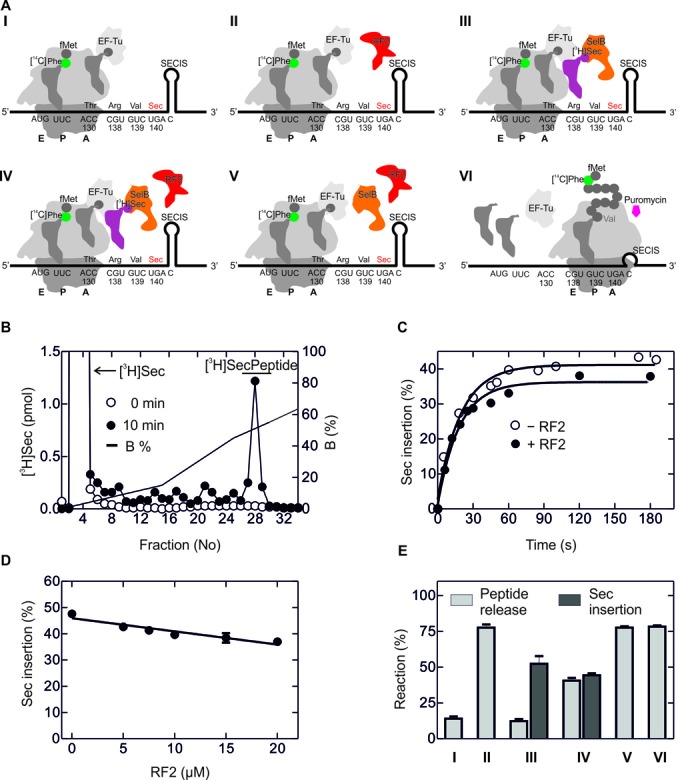
Sec insertion and RF2-mediated termination upon translation of *fdhF*-mRNA fragment. (**A**) Schematic of the experiments. I. Translation up to the UGA codon. Purified ribosome complexes programmed with the *fdhF* model mRNA and carrying fMet[^14^C]Phe-tRNA^Phe^ in the P site were mixed with aa-tRNA–EF-Tu–GTP complexes and EF-G–GTP. II. Termination at the UGA codon. The experiment was done as in I, except that RF2 was added. III. UGA recoding by Sec incorporation in the absence of RF2. As in I, but with addition of [^3^H]Sec-tRNA^Sec^–SelB–GTP. IV. Recoding and termination in the presence of [^3^H]Sec-tRNA^Sec^–SelB–GTP and RF2. V. Termination in the presence of SelB–GTP, as in IV, but without Sec-tRNA^Sec^. VI. Control for the maximum amount of nascent chains which can be released. After translation in the absence of [^3^H]Sec-tRNA^Sec^–SelB–GTP and RF2, puromycin (1 mM) was added to completely release nascent chains. (**B**) Separation of free [^3^H]Sec from [^3^H]Sec-containing peptide by HPLC on RP8. Examples are shown immediately after mixing (0 min) and after 10 min of translation. (**C**) Time course of Sec incorporation in the presence and absence of RF2 (7 μM). (**D**) Sec insertion at increasing RF2 concentrations. Error bars are SEM from three experiments. (**E**) Peptide release and Sec insertion upon translation at the conditions shown in A.

As a model mRNA, we used the fragment of *fdhF* gene coding for amino acids 130–156 (Supplementary Figure S2B) (Materials and Methods). We prepared 70S initiation complexes using the purified *fdhF*-mRNA, fMet-tRNA^fMet^ and initiation factors IF1, IF2, IF3. To determine the amount of translationally active 70S ribosomes in the mixture, we added to the 70S initiation complex the ternary complex EF-Tu–GTP–[^14^C]Phe-tRNA^Phe^, EF-G and GTP, purified the resulting post-translocation complex by sucrose density centrifugation and quantified the number of ribosomes carrying fMet-[^14^C]Phe-tRNA^Phe^ by nitrocellulose filtration and radioactivity counting (38). Translation was initiated by mixing the post-translocation complexes with EF-Tu–GTP–aa-tRNA complex prepared with total aminoacylated tRNA, and EF-G–GTP, in the absence or presence of [^3^H]Sec-tRNA^Sec^–SelB–GTP and RF2 (Figure 5A). Sec insertion was analyzed by reversed phase HPLC (RP-8) monitoring [^3^H]Sec (Figure 5B). The choice of radio-labeling and purification strategies was dictated by the necessities to quantify the ribosomes that were active in initiation on one hand and to determine the amount of Sec incorporated into peptides on the other hand. As expected, the amount of Sec-containing product, a 13 amino acid-long peptide, fMet-[^14^C]Phe-Thr-Asn-Asn-Val-Asp-Cys-Cys-Ala-Arg-Val-[^3^H]Sec, increased with time and reached a maximum after 1 min (Figure 5C). About 35–40% of the ribosomes incorporated [^3^H]Sec into peptides. Strikingly, the addition of a large excess of RF2 (7 μM) had essentially no effect on Sec insertion (Figure 5C). As the cellular concentration of RF2 may vary between 3 and 12 μM (23), we measured Sec insertion at increasing concentrations of RF2; however, at any RF2 concentration, the Sec insertion efficiency was >35% (Figure 5D).

To validate that RF2 can act on ribosome complexes with the SECIS in a native context, we tested the ability of RF2 to hydrolyze f[^3^H]Met-peptidyl-tRNA^Val^ bound to the P site after translation of the *fdhF* mRNA in the absence of Sec-tRNA–SelB–GTP (Supplementary Figure S3A) (Materials and Methods). The HPLC retention time of the peptide of the correct length was determined in experiments with [^14^C]Val in addition to f[^3^H]Met (data not shown). Because the peptide contains two Val residues, the ratio between [^14^C]/[^3^H] in the peptide should be 2:1, which unambiguously identified the peptide of correct length.

In the following experiments only the f[^3^H]Met label was used, because the presence of excess [^14^C]Val would interfere with the following analysis of the peptides released by RF2. The presence of RF2 did not affect the synthesis of the peptide up to the stop codon (Supplementary Figure S3B and C). To analyze the termination products with and without RF2, we took the translation mixture and analyzed the ratio of f[^3^H]Met in intact peptidyl-tRNA (before termination) and in released peptides (the products of termination). Samples were treated with salt to release peptidyl-tRNAs from ribosomes. Peptides (released spontaneously or by RF2-promoted termination) were separated from peptidyl-tRNA on a MonoQ column. In the absence of RF2, most of the peptide remained attached to the tRNA (Supplementary Figure S3D). When RF2 was added, increasing amounts of released peptides appeared with time (Supplementary Figure S3E). Quantification of the ratio between released and total peptide indicated that RF2 was capable of releasing ∼80% of the FdhH peptide after ∼2 min of translation, whereas in the absence of RF2 <20% was released (Supplementary Figure S3F). These results show that RF2 can terminate translation at a UGA codon in the context of the *fdhF* mRNA when SelB and Sec-tRNA are absent.

To compare the efficiencies of Sec insertion and RF2-dependent termination side by side, we measured overall translation, [^3^H]Sec insertion and [^14^C]-labeled nascent peptide in the same sample. From the fraction of [^14^C]Phe in the polypeptide we calculated that ∼75% of ribosomes engaged in translation at any condition, regardless of the addition of Sec-tRNA^Sec^–SelB–GTP or RF2. As expected, in the absence of RF2, only a small portion (15%) of the ribosomes spontaneously released peptides (Figure 5E, column I). Upon addition of RF2, most of translated peptide was released (column II). When Sec-tRNA^Sec^–SelB–GTP was added but no RF2, Sec was inserted on ∼40% of the ribosomes, whereas peptide release remained at a level of ∼15%, the same as in the absence of RF2 (column III). When additionally RF2 was present, the efficiency of Sec insertion was the same as in the absence of RF2 (column IV), suggesting that RF2 did not compete with Sec insertion. Termination efficiency was ∼35%; given that the maximum termination efficiency was 80%, RF2 appears to terminate translation on those ribosomes that did not incorporate Sec. In the presence of RF2 and SelB alone (without Sec-tRNA^Sec^), all ribosomes terminated translation at UGA and released the peptide (column V), to the same extent as in the control experiment where the peptide was released by the reaction with puromycin (column VI). Thus, the results obtained in a fully reconstituted *in vitro* translation system indicate that RF2 can efficiently bind to the UGA stop codon and promote termination when Sec-tRNA^Sec^–SelB–GTP is absent. However, in the presence of SelB, RF2 is capable of releasing peptides only on those ribosomes that were unsuccessful in incorporating Sec. Thus, RF2 turns out to be a very weak (if at all) inhibitor of Sec insertion.

## DISCUSSION

### Efficiency of Sec insertion *in vivo*

The present data show that the efficiency of selenoprotein synthesis *in vivo* is much higher than is usually assumed. In contrast to previous reports that suggested a very low recoding efficiency (∼5%) (21), we obtain a robust yield of Sec incorporation in the range of 30–40%. Visual analysis of the published results of Mansell *et al*. also suggests that UGA can be more efficiently recoded than usually assumed, namely to ∼25–60% (22). The most plausible reason for the difference in the reported values is the low concentration of selenium (1 μM) used in the earlier work, which appears too low compared to the concentration required to reach the maximum of Sec incorporation in our experiments (>10 μM Na_2_SeO_3_). We note that such high selenium concentrations are probably not needed for the synthesis of the endogenous bacterial selenoproteins; furthermore, the optimal concentration may depend on the reporter construct used. For example, cells grown anaerobically in the presence of 1 μM Na_2_SeO_3_ produced sizeable amounts of FdhH (50 mg protein from 300 g cells) (39). An early report suggested that saturation of UGA recoding in an *fdhF-lacZ* reporter construct occurred already at 0.1 μM selenium (10). The translation rates of native selenoproteins-coding mRNAs in *E. coli* are relatively low (40); thus even small amounts of a selenium source might be sufficient to produce full-length selenoproteins. However, the reporter constructs used by us and others are usually carried by mid-copy plasmids optimized for high levels of mRNA and protein production. It is possible that when the concentration of the respective mRNAs is high, the turnover of the Sec machinery may not be sufficient to maintain the amounts of Sec-tRNA^Sec^–SelB–GTP complex necessary to decode UGA on every mRNA, resulting in premature termination and, as a consequence, in an underestimation of the recoding efficiency. This observation is particularly important to take into account when expressing designer proteins containing Sec. Our finding that an excess of selenium source increases the incorporation efficiency, whereas the overexpression of tRNA^Sec^ has only little effect (21,22), suggests that the turnover may be limited by an enzyme that has a low affinity for selenium, e.g. by SelD which has a *K*_M_ value for selenide of 46 μM (41). Alternatively, because at selenite concentrations >1 μM selenium can be metabolized along the routes of sulfur metabolism (6), we cannot entirely exclude that the increased Sec incorporation in our experiment is due to indirect effects; however, such an explanation seems unlikely given that the effect is SECIS-dependent.

Earlier results obtained with *luc*–*fdhF*–*lacZ* reporter constructs suggested effects of the growth rate on Sec insertion ranging from 25 to 60% in rapidly and slowly growing cells, respectively (22). We were not able to reproduce this effect with our dual-luciferase reporter construct. Rather, we observed a very large effect of growth rate conditions on the overall protein production. After correction for the altered protein concentrations, the efficiency of Sec incorporation reached ∼40%, independent of growth rates. Also in this case, the difference to the earlier results may be attributed to different selenium concentrations, i.e. 50 μM (this paper) versus 1 μM Na_2_SeO_3_ (22). At low selenium concentration, the turnover capacity of the Sec machinery may control the yield of UGA recoding: when the rate of translation is high, Sec insertion may be limited, whereas at conditions of slow growth the turnover capacity might be sufficient even when the selenide concentration is low.

### Recapitulating the Sec-dependent translation *in vitro*

Many unresolved questions in understanding Sec-dependent recoding can possibly be answered by using an *in vitro* system that recapitulates the efficiency of Sec insertion *in vivo*. We used a translation system reconstituted from purified initiation and translation factors and a fragment of the *fdhF* mRNA coding for fMet, Phe and 10 amino acids from FdhH (amino acids 130–140), including UGA at position 140 and the SECIS. We systematically controlled the efficiency of initiation, elongation and Sec incorporation steps. While the efficiencies of initiation and elongation are high (∼75–80%), only ∼40% of actively translating ribosomes that reach the UGA codon insert Sec, even in the absence of RF2 and in the presence of excess Sec-tRNA^Sec^–SelB–GTP complexes over the ribosomes. The efficiency of UGA recoding by Sec *in vitro* is in excellent agreement with the results obtained *in vivo*. However, while the incomplete recoding *in vivo* can be due to competition with RF2 or other effects, as discussed below, the 40% Sec insertion efficiency *in vitro* obtained with an excess of active Sec-tRNA^Sec^–SelB–GTP ternary complexes (19) strongly indicates that Sec-tRNA^Sec^ delivery to the ribosome is inherently inefficient. Why some translating ribosomes incorporate Sec and others do not is currently unknown. UGA recoding by Sec is slow, leading to a long pause at the stop codon (21,40). One potential scenario is that half of the ribosomes lose the nascent peptide (or peptidyl-tRNA) during pausing. However, our analysis of translation, Sec insertion and RF2-mediated release suggest that this is unlikely, because (i) there is no spontaneous release of peptides, and (ii), when RF2 is added, the sum of Sec-containing peptide and peptide released by RF2 add up to the total amount of translated polypeptide, suggesting that all nascent chain complexes are functionally active. A more likely explanation is that Sec insertion is intrinsically inefficient owing to aggressive proofreading, inefficient Sec-tRNA^Sec^ accommodation or poor reactivity of Sec in the peptidyl transferase reaction. Future detailed kinetic experiments will clarify why—in contrast to other aminoacyl-tRNAs—the insertion of Sec-tRNA^Sec^ is not 100% efficient.

### Effect of RF2

Given that Sec insertion is intrinsically inefficient, the question arises what the role of RF2 is. *In vivo*, a large (12-fold) increase in RF2 concentration reduced specific UGA read-through by only 2.5-fold. Similarly, a 3–5-fold RF2 overexpression resulted in less than a 2-fold reduction of Sec insertion (22). These effects are surprisingly small, particularly given that the concentration of Sec-tRNA^Sec^–SelB–GTP in the cell is quite low (SelB 0.55 μM, tRNA^Sec^ 0.11 μM), compared to that of RF2 (3–12 μM (23)). Thus, *in vivo* Sec-tRNA^Sec^ is strongly preferred over RF2 when binding to a UGA codon followed by a SECIS element; As we show here, in comparison RF2 is a weak, if at all, competitor.

We note that many factors can modulate the ratio between Sec insertion and termination *in vivo*. Apart from the inherent inefficiency of Sec delivery to the ribosome or the potential competition with RF2, translation initiation may have an (indirect) effect on recoding. An optimal mRNA translation initiation region facilitates access of ribosomes to the start codon and thus accelerate ribosome loading. As a result, the distance between ribosomes translating *fdhF* mRNA may not provide enough time for the SECIS to refold between the passages of consecutive ribosomes. The *in vitro* efficiency of initiation on the native translation initiation region of *fdhF* mRNA is low (40%, compared to 80% on an optimized sequence, data not shown). On the other hand, profiling data indicate that ribosomes piled up upstream of the UGA codon (40); thus, it is possible that some ribosomes terminate at UGA, rather than incorporating Sec, because the SECIS did not refold after the passage of the preceding ribosome. In this scenario, the residual apparent effect of RF2 on Sec incorporation *in vivo* may result from the lacking SECIS, rather from the competition between termination and Sec insertion. Finally, stalling at a UGA codon with a SECIS at the mRNA entry to the ribosome may elicit cellular quality control responses that down-regulate translation, such as cleavage of the mRNA and induction of the *trans*-translation process, by analogy to the recently discovered regulatory effects of repetitive palindromic sequences (42), thereby additionally reducing the yield of Sec incorporation.

*In vitro*, when translating the longer *fdhF* mRNA, the efficiency of UGA recoding by Sec was ∼40% and RF2 had essentially no influence on Sec incorporation. Rather, RF2 had access to the UGA codon on the fraction of ribosomes that did not bind Sec. This suggests that Sec insertion at the UGA+SECIS sequence is strongly prioritized and escapes the competition with RF2; however, if Sec insertion fails, for instance due to slow accommodation and premature dissociation of Sec-tRNA^Sec^, RF2 gets a chance to access the stop codon and cause termination. In contrast to the long mRNA construct, on the shorter model mRNA with the coding sequence AUG UGA followed by the SECIS, Sec insertion was abolished when RF2 was added, although in the absence of RF2 the efficiency of Sec incorporation was as high as on the longer mRNA construct. With the shorter model construct, only the upper part of the stem loop is available for SelB recruitment when UGA has reached the decoding site, and the stop codon is accessible for either the Sec-complex or RF2 interaction. In this situation, Sec-tRNA^Sec^–SelB–GTP or RF2 are in direct competition for binding to the stop codon, and RF2 appears to be a much better ligand for the UGA, diminishing Sec insertion.

**Figure 6.**
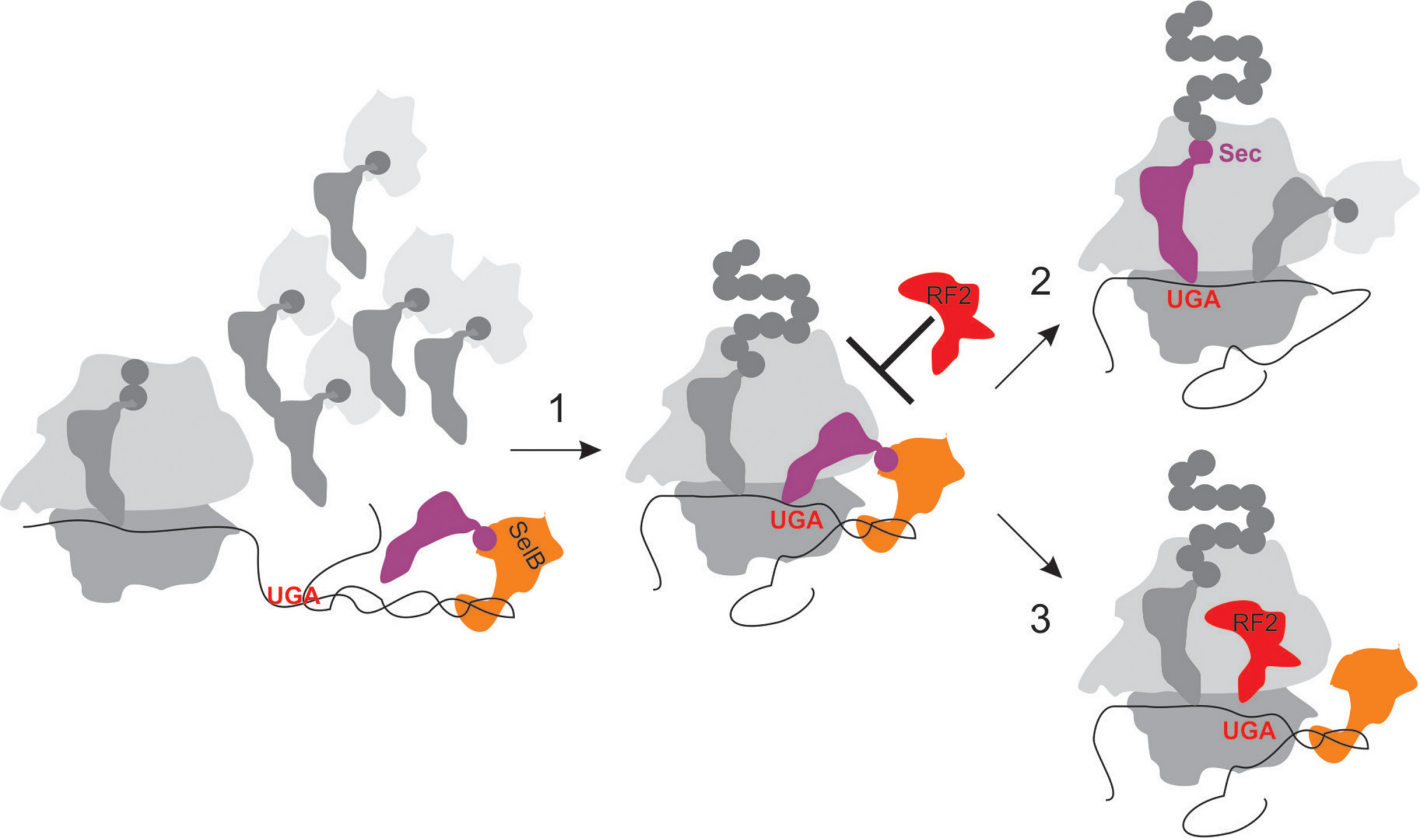
Model of SECIS-mediated Sec insertion versus RF2-dependent termination at UGA. Sec-tRNA^Sec^–SelB–GTP complex is rapidly recruited to the SECIS while still distant from the ribosome. P and A, tRNA-binding sites of the ribosome; 30S and 50S, ribosomal subunits. Step 1, during translation, the ribosome moves toward the UGA codon, the lower part of the SECIS becomes unwound, and the Sec-tRNA^Sec^–SelB–GTP complex, which is stabilized by the interaction with the SECIS prevents RF2 from entering the A site. Step 2, after delivery of Sec-tRNA^Sec^ to the A site and Sec insertion into the growing peptide, the ribosome can continue translation. Alternatively (step 3), if Sec delivery turns out unsuccessful, the A site becomes accessible for RF2 which promotes termination and peptide release.

At the onset of translation of the longer mRNA the ribosome occludes ∼11 nt within the mRNA tunnel (43). Thus, the SECIS element is located outside of the ribosome and is accessible for the interaction with Sec-tRNA^Sec^–SelB–GTP complex. SelB binds to the SECIS very rapidly (estimated to 1 μM × 10^8^ M^−1^ s^−1^ = 100 s^−1^ for the concentration of SelB used here and the reported association rate constant with the full-length SECIS (20), respectively). Such early recruitment may occur when the full-length SECIS is still distant from the ribosome (Figure 6). When the ribosome arrives at the UGA, the SECIS-bound Sec-tRNA^Sec^–SelB–GTP may block the entrance of RF2 to the A site. In this scenario, RF2 cannot compete with Sec-tRNA^Sec^, because dissociation of Sec-tRNA^Sec^–SelB–GTP from the SECIS is slow (20). However, if the attempt to deliver Sec is unsuccessful, the interaction with the SECIS may be lost, thereby freeing the access for RF2. Alternatively, conformational heterogeneity of translating ribosomes and the dynamics of the SECIS may define the preference for Sec binding on one fraction of ribosome complexes, whereas the other fraction favors RF2. In summary, our data suggest that RF2 does not act as a direct competitor of Sec, but rather terminates translation on those ribosomes where one round of Sec incorporation turned out to be unsuccessful.

## SUPPLEMENTARY DATA

Supplementary Data are available at NAR Online.

SUPPLEMENTARY DATA
