# Supplementary material for: Partitioning between recoding and termination at a stop codon–selenocysteine insertion sequence
Source: Nucleic Acids Res. 2015 Jun 3;43(13):6426–38. doi: 10.1093/nar/gkv558 (PMC4513850; doi:10.1093/nar/gkv558)
Supplement: SUPPLEMENTARY DATA [file supp_43_13_6426__index.html]

Partitioning between recoding and termination at a stop codon–selenocysteine insertion sequence — Partitioning between recoding and termination at a stop codon–selenocysteine insertion sequence — SUPPLEMENTARY DATA 

# Partitioning between recoding and termination at a stop codon–selenocysteine insertion sequence

## SUPPLEMENTARY DATA

- SUPPLEMENTARY DATA
