# Supplementary material for: Partitioning between recoding and termination at a stop codon–selenocysteine insertion sequence
Source: Nucleic Acids Res. 2015 Jun 3;43(13):6426–38. doi: 10.1093/nar/gkv558 (PMC4513850; doi:10.1093/nar/gkv558)
Supplement: SUPPLEMENTARY DATA [file supp_gkv558_nar-01032-m-2015-File008.docx]

**Supplementary Figures**

**
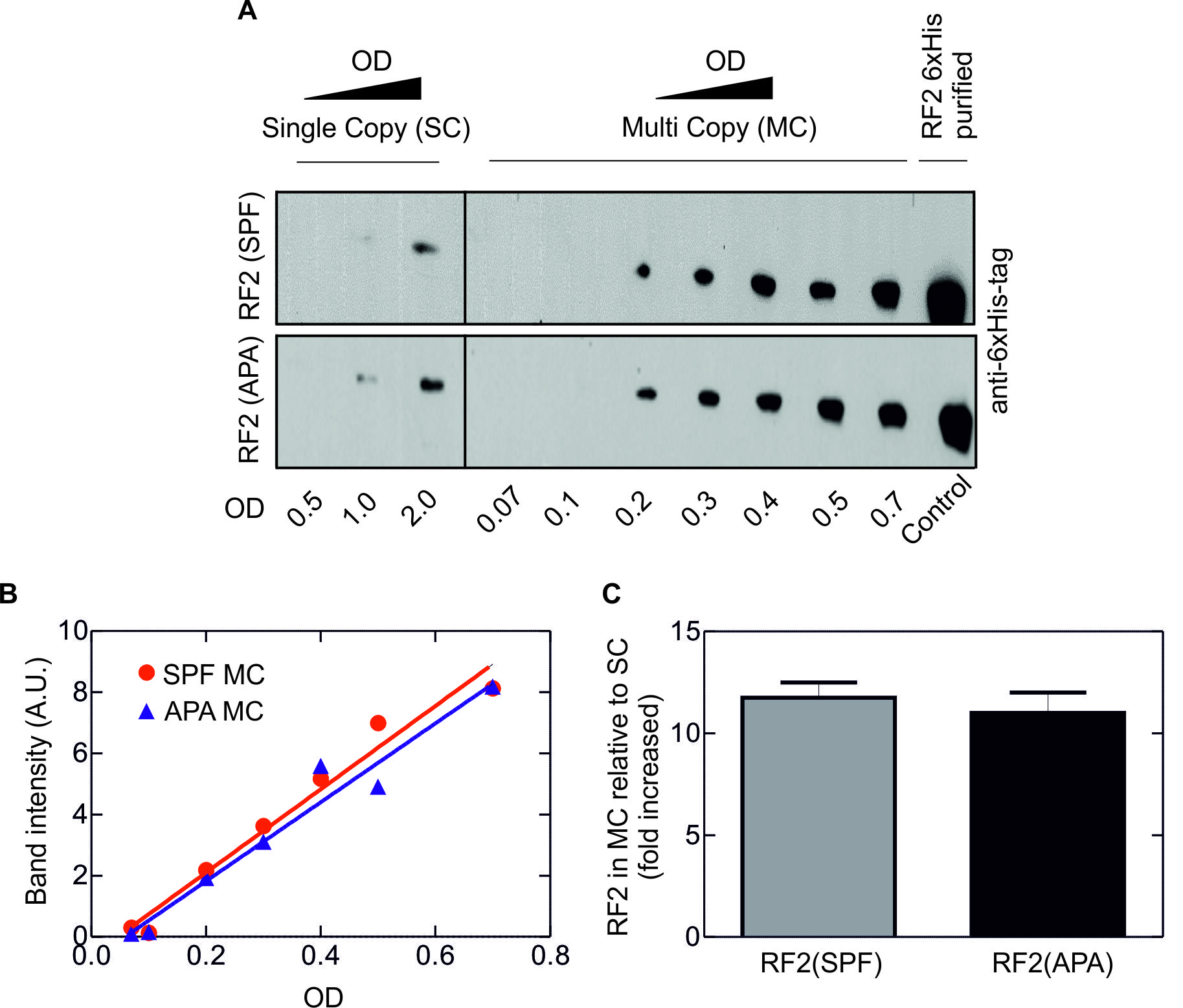
**

**Supplementary Figure S1.** RF2 expression at SC and MC conditions. (A) Western blot analysis using an antibody against 6xHis-tag. Different amounts of cells (OD_600_) containing RF2 (6xHis-tag) expressed at SC or MC conditions were lysed, proteins separated by SDS-PAGE and RF2 visualized using an antibody against the His-tag. As a control , purified 6xHis-tag RF2 was loaded. (B) Quantification of the Western blot band intensity for RF2(SPF) and RF2(APA) showing the linearity of the titration. (C) Overexpression of RF2 at MC over SC conditions calculated from the band intensities (for visual inspection, compare SC OD 2.0 and MC OD 0.2 and 0.3).

**
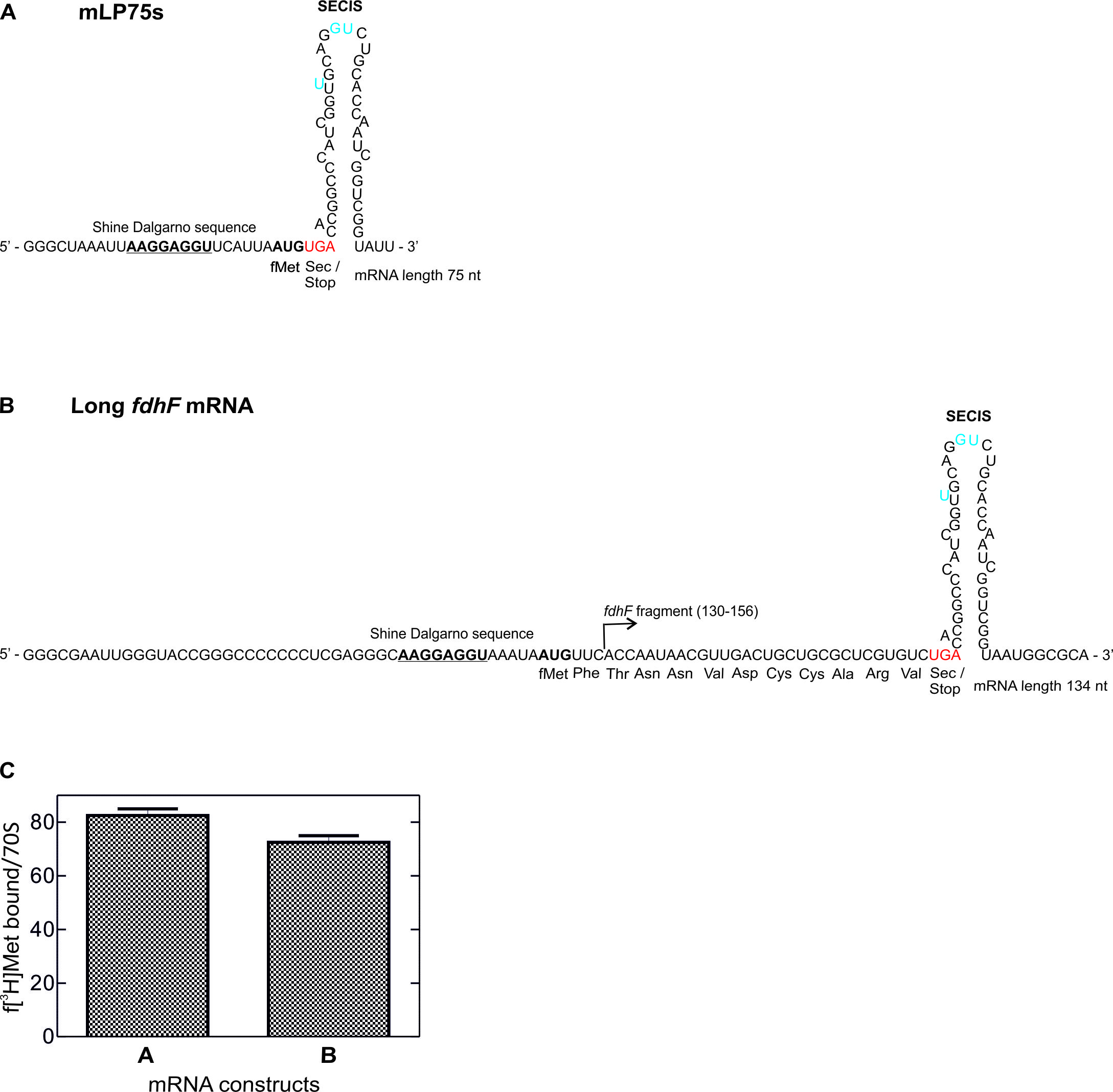
**

**Supplementary Figure S2.** mRNA constructs used for *in-vitro* translation. (A) Short model mRNA construct coding for AUG, UGA, and the natural SECIS of *fdhF* ([1](#_ENREF_1)), analogous to AH75 ([2](#_ENREF_2)). (B) A model mRNA construct with a coding sequence for fMet-Phe plus amino acids 130-156 of FdhF, with the UGA codon at position 140 followed by the SECIS. (C) Initiation efficiency of the two model mRNAs, as determined by nitrocellulose filtration, and measuring f[^3^H]Met-tRNA^fMet^.

**
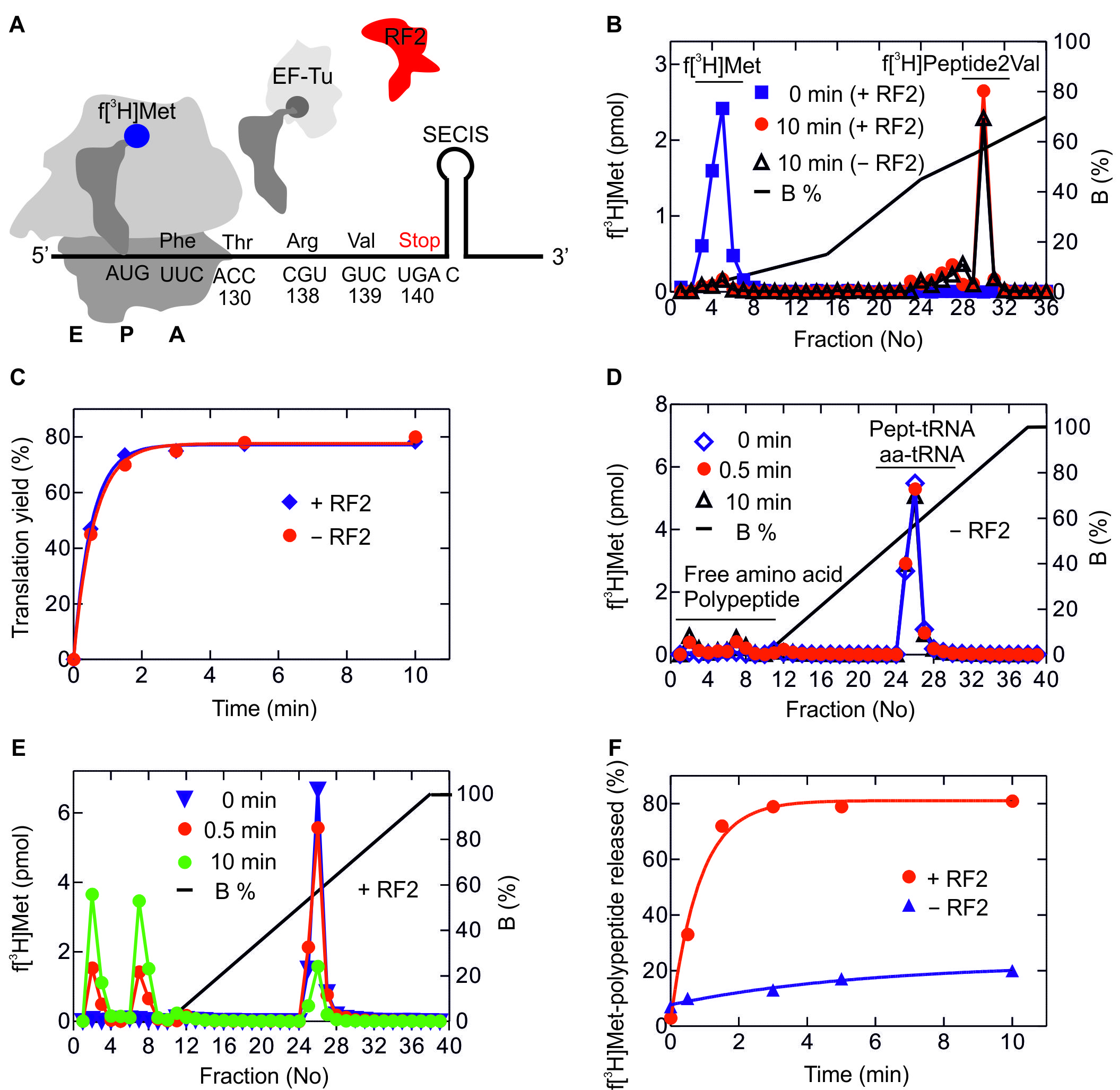
**

**Supplementary Figure S3.** Analysis of peptide release on the *fdhF* mRNA. (A) Schematic of experiment. Single-turnover *in-vitro* translation up to the second Val codon in the *fdhF* mRNA followed by RF2-dependent termination. (B) Separation of free f[^3^H]Met from the FdhH peptide by HPLC on an RP8 column. The duration of translation is indicated in the inset. The desired length of the peptide containing two Val residues was confirmed in independent runs using [^14^C]Val (not shown). (C) Yield of translation relative to the amount of initiation complexes. (D) Analysis of peptidyl- (and aminoacyl-, aa) tRNA after translation in the absence of RF2 for the times indicated in the inset. Ribosomes were salt-washed to set free tRNAs, and the material was applied to a MonoQ column. Free peptides (and amino acids) were in the flow-through; peptidyl- and aa-tRNAs were retained on the column and eluted with high salt buffer. The two peaks in the flow-through are due to two sequential injections of the sample to the column. (E) Same as E, but in the presence of RF2. (F) Quantification of peptide release after *fdhF* mRNA translation in the absence (blue symbols) and presence (red symbols) of RF2 (0.4 µM), all in the absence of Sec-tRNA^Sec^–SelB–GTP.

**Supplementary References**

1. Fischer, N., Paleskava, A., Gromadski, K.B., Konevega, A.L., Wahl, M.C., Stark, H. and Rodnina, M.V. (2007) Towards understanding selenocysteine incorporation into bacterial proteins. Biol Chem, 388, 1061-1067.

2. Huttenhofer, A. and Bock, A. (1998) Selenocysteine inserting RNA elements modulate GTP hydrolysis of elongation factor SelB. Biochemistry, 37, 885-890.
